# Investigation of the Effectiveness of Traditional Breathing Therapy on Pulmonary Function in College Students with Obstructive Sleep Apnea

**DOI:** 10.1155/2022/1674973

**Published:** 2022-07-15

**Authors:** Yunxiang Pei, Yongzhao Fan, Xiaoyang Kong, Huan Sun, Jun Zhou, Hao Wu

**Affiliations:** ^1^Capital University of Physical Education and Sports, 100191, Beijing, China; ^2^Comprehensive Key Laboratory of Sports Ability Evaluation and Research of the General Administration of Sport of China, Beijing Key Laboratory of Sports Function Assessment and Technical Analysis, Capital University of Physical Education and Sports, Beijing 100191, China

## Abstract

**Background:**

Obstructive sleep apnea (OSA) is a problem that involves many body systems, but its impact on the respiratory system deserves special attention. While there are many studies investigating the use of continuous positive airway pressure (CPAP) to treat lung function in patients with sleep apnea, the lack of studies in the literature on the effects of traditional breathing therapy on lung function in patients with OSA prompted us to conduct such a study.

**Objective:**

The present randomized trial aims to assess the effect of traditional breathing therapy on daytime sleepiness and pulmonary function in college students with OSA.

**Methods:**

Forty college students (male) with OSA were randomly divided into two groups: the control group (CG) and the traditional breathing therapy group (TG). Daytime sleepiness symptoms in OSA are measured primarily by the Epworth Sleepiness Scale (ESS). Pulmonary function measurements included FVC, FEV1, PEE, and MEF50%. The changes in morning blood pressure (BP), including diastolic BP and systolic BP, were also recorded. Data were recorded before and after the experiment.

**Results:**

A decrease in ESS at 12 weeks after intervention had statistical significance compared with values recorded before intervention (*P* < 0.05). A decrease in systolic and diastolic BP at 12 weeks after intervention had statistical significance compared with values recorded before intervention (*P* < 0.05). Comparisons made in terms of pulmonary functions demonstrated a statistically significant increase in 12-week postintervention values of FVC, FEV1, PEF, and MEF50% (*P* < 0.05).

**Conclusion:**

Our study shows the positive effects of traditional breathing therapy on pulmonary function parameters. This suggests that traditional breathing therapy treatment in OSA patients is as effective as CPAP on pulmonary function, while there is an improvement in daytime sleepiness and a modest decline in the mean daytime systolic and diastolic BP.

## 1. Introduction

Most people will sleep for roughly a third of their lives, which is necessary for a variety of health tasks, including controlling homeostasis across many body and mental systems [1]. Adequate sleep is necessary for a variety of immediate physiological and behavioral advantages, including immune system regulation, cardiorespiratory fitness, and tiredness prevention [1, 2]. Sleep is also important for long-term health, as it is thought to be involved in systems that combat chronic diseases including obesity, hypertension, metabolic syndrome, and even cancer [2–4]. Sleep is important not only for health but also for many aspects of cognition, including memory, executive functioning, attention, and emotional control, allowing people to perform at their best [5]. As a result, the cost of inadequate sleep might be significant.

OSA is a common disorder with an estimated incidence of 10–20% in the adult population [6, 7], and it can present with cognitive symptoms such as fatigue, difficulty concentrating, and depression in college students. Numerous studies have demonstrated an association between OSA and cardiovascular disease, chronic heart failure ischemia, hypertension, obesity, and impaired glucose tolerance [8, 9]. Many aspects of the pathophysiology, such as the pharynx, oropharynx, base of the tongue, and epiglottis, can cause upper airway obstruction, leading to OSA. The most prevalent causes of sleep apnea are obstructions at the base of the tongue and partial or total obstructions of the upper respiratory tract. The upper airway collapse characterizes during sleep due to decreased basal and compensatory upper airway muscle activities. Recurrent narrowing or collapse of the upper airway occurs during sleep and is associated with episodic hypoxemia, hypercapnia, increased respiratory effort, and disruption of sleep architecture. OSA is linked to a decline in quality of life as well as an increase in morbidity and death due to metabolic, neurological, and cardiovascular changes, according to mounting evidence [10–13]. OSA is a substantial risk factor for hypertension, myocardial infarction, stroke, and a reduction in patient quality of life [14].

Treatment for OSA has several advantages, including reduced daytime drowsiness, improved quality of life, lower blood pressure, and reduced cardiovascular morbidity [15–18]. The use of CPAP and oral appliances during sleeping, as well as surgery, are the most widely approved therapies in the treatment of OSA [19–21]. The gold standard for OSA therapy for the past 35 years has been nocturnal pressure support, such as CPAP, which serves to pneumatically splint the throat open. Through this treatment, OSA and its adverse reactions were effectively alleviated [22]. However, CPAP is intolerable for many patients, and alternative treatment methods are frequently sought [23–27]. Physical exercise and rehabilitation are two more possibilities for helping OSA patients. Aerobic activities are aimed at cardiovascular rehabilitation [28], and rehabilitation exercises focus on the orofacial region and breath training [29, 30]. The lessening of breathing issues during sleep has been mentioned as an impact of aerobic activities. Cardiovascular rehabilitation was found to be additive to CPAP in several trials [31]. Exercise is considered a nonpharmacological therapeutic option for sleep disorders by the American Sleep Apnea Association [32]. The orofacial myofunctional therapy (OMT), or oropharyngeal exercises, was presented with efficacy for lowering OSA severity and associated symptoms in adults, with one of the key areas in the promotion of changes in dysfunctional upper airway muscles [30]. The traditional breathing therapy is similar to OMT. The advantage of traditional breathing therapy is that the therapy is easy to learn, easy to operate, does not require wearing any equipment, and is done by hand. Unlike CPAP, which requires wearing a mask, which leads to poor patient compliance, patients cannot be allocated for a long time, and the treatment effect is not good.

Although some studies have examined the use of CPAP to treat pulmonary function parameters in patients with OSA [33, 34], it is worth noting that there are currently no studies in the literature investigating the effects of traditional breathing therapy for OSA on pulmonary function. Our study aims to investigate the efficacy of traditional breathing therapy on lung function in patients with OSA and to demonstrate the positive effects of traditional breathing therapy on lung function, similar to the effects of CPAP.

## 2. Methods

### 2.1. Subjects

The Ethics Committee of the Capital University of Physical Education and Sports approved the study (2019A44). The inclusion criteria for this study included 40 college students (male) with good general health (stable outpatient status) with OSA symptoms (snoring, breathing cessation, and daytime sleepiness). Forty college students with OSA were randomly divided into control groups (CG, subjects = 20) and traditional breathing therapy groups (TG, subjects = 20). College students in the TG group underwent traditional breathing therapy training for 12 weeks. The control group has had no traditional breathing therapy training for 12 weeks. Subjects suitable for inclusion criteria were asked if they had the habit of smoking and exercising. Anthropometric data, lung functions, ESS, and blood pressure were repeatedly measured at the end of week 12 to describe the effects of traditional breathing therapy treatments in college students with OSA. Written informed consent was obtained from all participants. Baseline laboratory data are listed in Table 1.

### 2.2. Protocol

The subjects in the TG group were treated with traditional breathing therapy in the morning and evening and 10 minutes each time, and the intervention period was 12 weeks. Traditional breathing therapy was taught to the patients repetitively until they said “I understood.” TG group includes the exercise of the buccal site, throat, and neck in cooperation with breathing. The traditional breathing therapy interventions are done independently by the participant and are based on video recordings made during each traditional breathing therapy intervention, with videos of the breathing therapy exercise collected once a week. The content of traditional breathing therapy mainly includes the following:

① Acupoint massage: BingTong, Yingxiang, Tiantu, Tianhu.
② Roll the tongue and press the upper palate with force, close your eyes and meditate, and breathe thoroughly. To cooperate with your breathing, roll your tongue against your upper jaw when you inhale and relax when you exhale. Exhale and inhale like this; repeat 60 times.
③ Sweep the gums: press the tongue against the inner gums, nine times clockwise and counterclockwise and then against the outer gums, nine times clockwise and counterclockwise.
④ Three pharyngeal body fluids: when the saliva is whole, swallow it three times, making as much noise as possible when swallowing until it reaches the Dantian. Then, puff up your cheeks with air and then retract them. Bulge when exhaling, relax when inhaling, and repeat 60 times.
⑤ Extend your tongue forward and retract your cheeks: close your eyes, meditate, and breathe thoroughly. Then, suddenly, open your mouth wide, stick your tongue forward as much as possible, and at the same time, retract your lips and cheeks and fully stretch the base of your tongue. With breathing, extend when inhaling and retract when exhaling. Repeat these 18 times.
⑥ Neck stretching exercise: sit or stand, hands-on-hips, eyes closed, and breathe thoroughly. First, do the left and right neck extension. When inhaling, stretch the head and neck as far as possible to the upper left side, when exhaling, return to the neutral position, when inhaling again, stretch the head and neck as far as possible to the upper right side, and then retract it when exhaling. Repeat these 18 times. Then, do back-up and neck-lifting movements, front-bottom bowing, and neck-lifting movements, breathing 18 times.

### 2.3. Test

#### 2.3.1. Filling Basic Information

All tests were conducted in a laboratory that are required to be quiet and bright. Before the tests, all subjects were told about this experiment's purpose and concrete steps. After agreeing to participate in the investigation, they filled in the basic information, including name, sex, age, major, and exercise history.

#### 2.3.2. Body Composition Test

Body composition was tested by a body composition analyzer (Genius-220). Everyone stood on the testing platform without shoes. After measuring body weight, they entered their height, age, and sex into the analyzer in order and then the test began. They need to hold the handle at arm's length, lean the body forward slightly, and keep this position until the test finished. The main indicators we acquired were height, weight, and body type.

#### 2.3.3. Daytime Sleepiness Evaluation

The Turkish Epworth Drowsiness Scale was used to measure subjective sleepiness (ESS). ESS is a patient-reported questionnaire that assesses situational sleep propensities to detect excessive daytime sleepiness (EDS) (Johns, 1991). Specifically, the ESS consists of eight items that assess the likelihood of falling asleep in real-world situations, such as reading, watching television, or driving. Each item is graded on a scale of zero to three, giving a total score of zero to 24, with higher values indicating more severe EDS. Values of ten or less are considered normal, whereas scores of ten or more indicate abnormal EDS (Johns, 1997) [35].

#### 2.3.4. Pulmonary Function Test

The subjects' lung functions were assessed using pulmonary function testing. An expert used a COSMED Micro Quark machine to do spirometry (COSMED, Italy). FVC, FEV1, PEE, and MEF50% values were recorded.

#### 2.3.5. Blood Pressure

Blood pressure testing was performed using a standard mercury sphygmomanometer, subjects were asked to sit still for one minute before the test, and the test was performed when calm.

### 2.4. Statistical Analysis

Data were analyzed by SPSS25.0 (SPSS Inc., Chicago, IL, USA). The results were expressed as means ± SD, and all data showed normal distribution. Experimental data results were compared between baseline and later time-points using ANOVA. *p* < 0.05 was considered statistically significant.

## 3. Results

Forty college students with OSA were randomized as exercise and control groups. Data were presented for the CG and TG. All of the 20 college students with OSA in the TG participated in the training. No problems occurred.

### 3.1. Characteristics of the Subjects

Table 1 shows the baseline characteristics of the participants in each group. Height, weight, and neck circumference did not differ significantly (*P* > 0.05).

### 3.2. ESS

**Figure 1:**
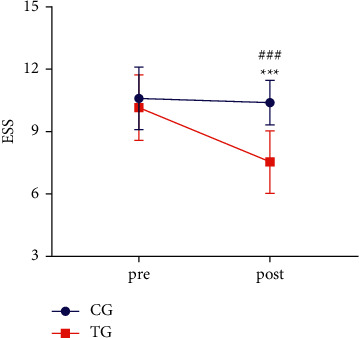
Comparison of ESS between the CG and TG groups. Pre, before the experiment. Post, after the experiment. Compared with CG (post), ^###^*P* < 0.001; compared with TG (pre), ^*∗∗∗*^*P* < 0.001.

Figure 1 is the ESS score in each group before and after the intervention.

At the baseline, there were no significant differences in ESS scores between the two groups (*P* > 0.05). The ESS scores were lower in the TG (post) 7.55 ± 1.50) than in the CG (post) (10.40 ± 1.07) (*P* < 0.001). The result revealed a significant improvement between the TG (pre) (10.15 ± 1.57) and TG (post) (7.55 ± 1.50) (*p* < 0.001). There was no significant difference among CG (post), CG (pre), and TG (pre) (*P* > 0.05).

### 3.3. Systemic Blood Pressure

**Figure 2:**
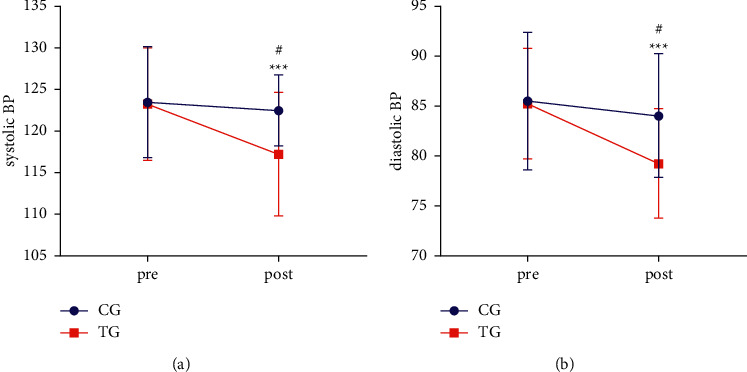
(a, b) Comparison of systemic blood pressure between the CG and TG group. Pre, before the experiment. Post, after the experiment. Compared with CG (post), ^#^*P* < 0.05, and compared with TG (pre), ^*∗∗∗*^*P* < 0.001.

Figure 2(a) shows the systolic BP in each group. Figure 2(b) shows the diastolic BP in each group.

Figure 2(a) shows the systolic BP in each group before and after the intervention. At the baseline, there were no significant differences in systolic BP between the two groups (*P* > 0.05). The systolic BP was lower in the TG (post) (117.20 ± 7.45) than in the CG (post) (122.50 ± 4.25) (*P* < 0.05). The result revealed a significant improvement between the TG(pre) (123.25 ± 6.74) and TG (post) (117.20 ± 7.45) (*p* < 0.001). There was no significant difference among CG (post), CG (pre), and TG (pre) (*P* > 0.05). Figure 2(b) shows the diastolic BP in each group before and after the intervention. At the baseline, there were no significant differences in diastolic BP between the two groups (*P* > 0.05). The diastolic BP was lower in the TG (post) (79.2 5 ± 5.45) than in the CG (post) (84.00 ± 6.15) (*P* < 0.01). The result revealed a significant improvement between the TG (pre) (85.25 ± 79.25) and TG (post) (79.25 ± 5.45) (*p* < 0.01). There was no significant difference among CG (post), CG (pre), and TG (pre) (*P* > 0.05).

### 3.4. Pulmonary Function Parameter Results

**Figure 3:**
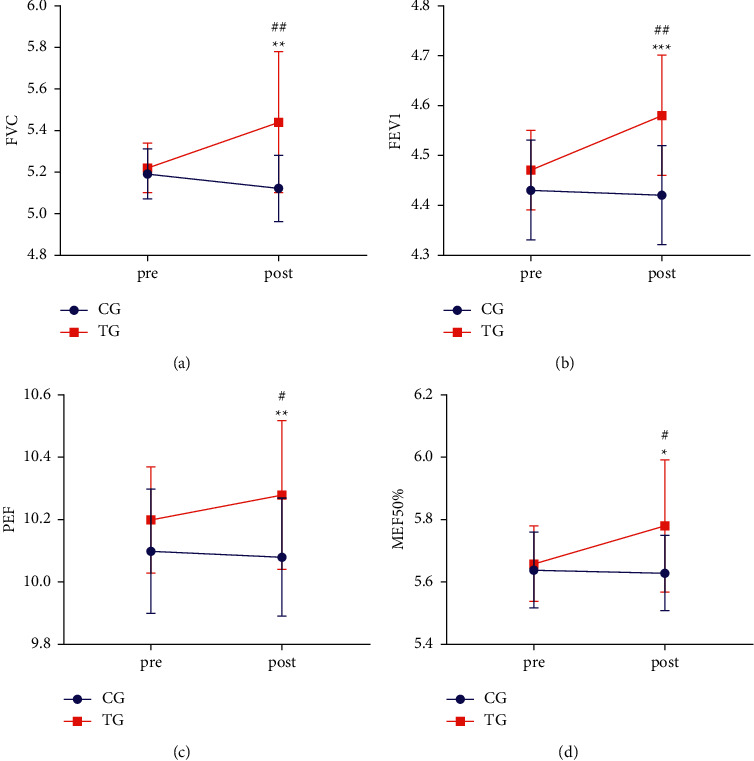
(a–d) Comparison of pulmonary function parameters between the CG and TG group. Pre, before the experiment. Post, after the experiment.

Figure 3(a) shows the FVC in each group. Compared with CG (post), ^##^*P* < 0.01, and compared with TG (pre), ^*∗∗*^*P* < 0.01. Figure 3(b) shows the FEV1 in each group. Compared with CG (post), ^##^*P* < 0.01, and compared with TG (pre), ^*∗∗∗*^*P* < 0.001. Figure 3(c) shows the PEF in each group. Compared with CG (post), ^#^*P* < 0.05, and compared with TG (pre), ^*∗∗*^*P* < 0.01. Figure 3(d) shows the MEF50% in each group. Compared with CG (post), ^#^*P* < 0.05, and compared with TG (pre), ^*∗*^*P* < 0.05.

Figure 3(a) shows the FVC in each group before and after the intervention. At the baseline, there were no significant differences in FVC between the two groups (*P* > 0.05). The FVC was higher in the TG (post) (5.44 ± 0.34) than in the CG (post) (5.12 ± 0.16) (*P* < 0.01). The result revealed a significant improvement between the TG (pre) (5.22 ± 0.12) and TG (post) (5.44 ± 0.34) (*p* < 0.01). There was no significant difference among CG (post), CG (pre), and TG (pre) (*P* > 0.05). Figure 3(b) shows the FEV1 in each group before and after the intervention. At the baseline, there were no significant differences in FEV1 between the two groups (*P* > 0.05). The FEV1 was higher in the TG (post) (4.58 ± 0.12) than in the CG (post) (4.43 ± 0.10) (*P* < 0.01). The result revealed a significant improvement between the TG (pre) (4.47 ± 0.08) and TG (post) (4.58 ± 0.12) (*p* < 0.001). There was no significant difference among CG (post), CG (pre), and TG (pre) (*P* > 0.05). Figure 3(c) shows the PEF in each group before and after the intervention. At the baseline, there were no significant differences in PEF between the two groups (*P* > 0.05). The PEF was higher in the TG (post) (10.28 ± 0.24) than in the CG (post) (10.08 ± 0.19) (*P* < 0.05). The result revealed a significant improvement between the TG (pre) (10.18 ± 0.17) and TG (post) (10.28 ± 0.24) (*p* < 0.01). There was no significant difference among CG (post), CG (pre), and TG (pre) (*P* > 0.05). Figure 3(d) shows the MEF50% in each group before and after the intervention. At the baseline, there were no significant differences in MEF50% between the two groups (*P* > 0.05). The MEF50% was higher in the TG (post) (5.78 ± 0.21) than in the CG (post) (5.63 ± 0.12) (*P* < 0.05). The result revealed a significant improvement between the TG (pre) (5.66 ± 0.12) and TG (post) (5.78 ± 0.21) (*p* < 0.05). There was no significant difference among CG (post), CG (pre), and TG (pre) (*P* > 0.05).

## 4. Discussion

OSA is a poorly understood and difficult-to-treat illness that is a leading source of serious health problems [36]. OSA is a very common respiratory condition [37, 38]. OSA is a condition marked by repeated partial or total blockage of the upper respiratory tract, resulting in sleep hypoxia.

OSA is defined as a condition in which the upper airway is repeatedly obstructed, resulting in reduced oxygen saturation, waking from sleep, excessive snoring, and increased daytime sleepiness [39]. Failure to maintain patency of the upper airway (UA) during sleep is one of the causes of OSA and its sequelae [40, 41]. There are three major variables that contribute to the development of upper airway obstruction: anatomical structure of the upper respiratory tract, the negative pressure generated during respiration, and inactivation of muscles that expand the pharyngeal airway [42]. OSA causes hypoxemia and hypercapnia due to nocturnal apnea, resulting in decreased oxygen saturation and affecting pulmonary function. Several previous studies have explored lung function in OSA patients [33, 43]. Furthermore, OSA may cause impairment of lung function and may lead to restrictive or occasionally obstructive pulmonary disease. OSA patients may develop respiratory mechanical disorders that adversely affect lung function, such as an increase in respiratory work and a decrease in lung volumes.

Traditional breathing therapy in this protocol mainly stretches the muscles of the cheeks, throat, and neck and completes it with breathing. This therapy coordinates the neck, face, jaw, throat, tongue, and mouth exercises. Traditional breathing therapy involves isotonic and isometric exercises aimed at oral and oropharyngeal structures, intending to increase pharyngeal and peripharyngeal muscle tone, endurance, and coordination [44–46]. By directly training the tongue and other pharyngeal muscles, traditional breathing therapy determines the reduction of tongue fat and neck circumference [46]. Exercise therapy stabilizes neuromuscular function and motor control of muscles in the upper airway. Thus, traditional breathing therapy helps reposition the tongue, improve nasal breathing, and increase muscle tone and responsiveness in pediatric and adult OSA patients [44, 45]. Improvements in nasal breathing and the concomitant reduction in nocturnal oral breathing seem to reduce OSA severity and snoring [47, 48].

CPAP therapy has been reported to improve ESS, mental vitality, and social function [49, 50]. Parallel to this study, we discovered a significant change in ESS after traditional breathing therapy. At the baseline, there were no significant differences in ESS scores between the two groups (*P* > 0.05). In the control group, this stayed unaltered, but in the exercise group, it changed. This study showed that the ESS after traditional breathing therapy intervention was significantly lower than that before treatment, indicating that traditional breathing therapy improved daytime sleepiness in snoring people. We felt that, as with traditional breathing therapy, efficient sleep occurred, and this boosted critical activities like job productivity and vigor (Figure 1).

Several studies have reported an immediate drop in intra-arterial blood pressure during nocturnal and morning wakefulness [51, 52] and a modest decrease in mean daytime systolic and diastolic blood pressure after CPAP treatment [53]. Parallel to this study, we also found a significant change in systolic BP and diastolic BP after traditional breathing therapy(Figure 2). This supports the hypothesis that there may be a direct link between OSA and systemic blood pressure control. Patients with OSA are unresponsive to bradykinin's venous dilation, which leads to the development of systemic hypertension and appears to be reversible with nasal CPAP therapy [54]. Similar to CPAP, traditional breathing therapy also has positive effects on the systemic BP of college students with OSA(Figure 2).

In our study, we measured the five fundamental measures (FVC, FEV1, PEF, and MEF50%) from a group of 20 students with OSA and found a considerable improvement after traditional breathing therapy. It has been shown that exercise can improve the FVC percentage and FEV1/FVC ratio [55]. Parallel to this study, we discovered that traditional breathing therapy resulted in a considerable rise in FEV1. In a study on the use of CPAP, it was suggested that there may be a relationship between lung capacity and OSA. In this study, OSA therapy with CPAP showed a substantial improvement in FEV1 values after 6 months [56]. On the other hand, FEV1 has been shown to have a predictive impact in OSA patients treated with nasal CPAP [57]. Our study shows a significant improvement in FEV1 after traditional breathing therapy. Similar to CPAP, traditional breathing therapy also has positive effects on pulmonary function (Figure 3).

## 5. Conclusions

OSA is a critical public health issue that can lead to serious chronic illnesses. Even though the causes of OSA are complicated, data show that inadequate upper airway muscle performance is a major factor in airway blockage. We developed an evidence-based traditional breathing therapy that improves muscle function in specific oropharyngeal and cervical-cheek muscle groups. In our study to evaluate pulmonary function before and after traditional breathing therapy in college students with OSA, significant improvements were obtained in all four postoperative parameters (FVC, FEV1, PEF, and MEF50%) in these results. This study demonstrates the positive effect of traditional breathing therapy on pulmonary function in treating college students with OSA. In our study, traditional breathing therapy treatment of OSA showed positive effects on pulmonary functions, similar to the effects of CPAP, and a modest decline in mean daytime systolic and diastolic BP. Traditional breathing therapy can be used as a convenient and applicable prevention or adjuvant treatment for OSA and can be incorporated into the existing treatment. However, further and broader research on this topic is warranted.

## Figures and Tables

**Table 1:** Descriptive characteristics of the 30 subjects.

	CG (*n* = 20)	TG (*n* = 20)
Age (years) mean (SD)	20.7(1.1)	20.8(1.3)
Weight (kg) mean (SD)	75.3(9.8)	73.4(7.8)
Neck circumference (cm) mean (SD)	34.9(3.4)	37.3(2.2)
Height (cm) mean (SD)	178.3(3.2)	178.6(3.6)

## Data Availability

Data can be obtained upon request to the corresponding author.
